# A remote digital memory composite to detect cognitive impairment in memory clinic samples in unsupervised settings using mobile devices

**DOI:** 10.1038/s41746-024-00999-9

**Published:** 2024-03-26

**Authors:** David Berron, Wenzel Glanz, Lindsay Clark, Kristin Basche, Xenia Grande, Jeremie Güsten, Ornella V. Billette, Ina Hempen, Muhammad Hashim Naveed, Nadine Diersch, Michaela Butryn, Annika Spottke, Katharina Buerger, Robert Perneczky, Anja Schneider, Stefan Teipel, Jens Wiltfang, Sterling Johnson, Michael Wagner, Frank Jessen, Emrah Düzel

**Affiliations:** 1 German Center for Neurodegenerative Diseases, Magdeburg, Germany; 2 Clinical Memory Research Unit, Department of Clinical Sciences Malmö, Lund University, Lund, Sweden; 3 neotiv GmbH, Magdeburg, Germany; 4 Institute for Cognitive Neurology and Dementia Research, Otto-von-Guericke University, Magdeburg, Germany; 5 Department of Medicine, Division of Geriatrics and Gerontology, University of Wisconsin, School of Medicine and Public Health, Madison, Wisconsin US; 6 Geriatric Research Education and Clinical Center, William S. Middleton Memorial Veterans Hospital, Madison, Wisconsin USA; 7 German Center for Neurodegenerative Diseases, Bonn, Germany; 8 German Center for Neurodegenerative Diseases, Munich, Germany; 9 Institute for Stroke and Dementia Research (ISD), University Hospital, LMU Munich, Munich, Germany; 10 Department of Psychiatry and Psychotherapy, University Hospital, LMU Munich, Munich, Germany; 11 Munich Cluster for Systems Neurology (SyNergy), Munich, Germany; 12 Ageing Epidemiology Research Unit (AGE), Imperial College London, London, UK; 13 Department for Neurodegenerative Diseases and Geriatric Psychiatry, University Hospital Bonn, Bonn, Germany; 14 Department of Psychosomatic Medicine, Rostock University Medical Center, Rostock, Germany; 15 German Center for Neurodegenerative Diseases, Rostock, Germany; 16 German Center for Neurodegenerative Diseases, Göttingen, Germany; 17 Department of Psychiatry and Psychotherapy, University Medical Center Göttingen, University of Göttingen, Göttingen, Germany; 18 German Center for Neurodegenerative Diseases, Cologne, Germany

**Keywords:** Alzheimer's disease, Diagnostic markers, Human behaviour

## Abstract

Remote monitoring of cognition holds the promise to facilitate case-finding in clinical care and the individual detection of cognitive impairment in clinical and research settings. In the context of Alzheimer’s disease, this is particularly relevant for patients who seek medical advice due to memory problems. Here, we develop a remote digital memory composite (RDMC) score from an unsupervised remote cognitive assessment battery focused on episodic memory and long-term recall and assess its construct validity, retest reliability, and diagnostic accuracy when predicting MCI-grade impairment in a memory clinic sample and healthy controls. A total of 199 participants were recruited from three cohorts and included as healthy controls (*n* = 97), individuals with subjective cognitive decline (*n* = 59), or patients with mild cognitive impairment (*n* = 43). Participants performed cognitive assessments in a fully remote and unsupervised setting via a smartphone app. The derived RDMC score is significantly correlated with the PACC5 score across participants and demonstrates good retest reliability. Diagnostic accuracy for discriminating memory impairment from no impairment is high (cross-validated AUC = 0.83, 95% CI [0.66, 0.99]) with a sensitivity of 0.82 and a specificity of 0.72. Thus, unsupervised remote cognitive assessments implemented in the neotiv digital platform show good discrimination between cognitively impaired and unimpaired individuals, further demonstrating that it is feasible to complement the neuropsychological assessment of episodic memory with unsupervised and remote assessments on mobile devices. This contributes to recent efforts to implement remote assessment of episodic memory for case-finding and monitoring in large research studies and clinical care.

## Introduction

Differentiating mild cognitive impairment (MCI) from subjective cognitive impairment is important to provide a prognosis regarding future cognitive decline as well as regarding the potential eligibility for treatments at the MCI stage of Alzheimer’s disease (AD). However, differentiating MCI from subjective cognitive impairment is still very challenging using brief cognitive tests^1^. More than 80% of older adults who seek medical advice due to memory complaints and who are later found to have a biomarker profile indicative of AD have an amnestic variant with predominant episodic memory impairment^1^. Indeed, episodic memory, the ability to recall spatial and temporal relationships of personally experienced events^2^, is a key component of the neuropsychological assessment of individuals with suspected AD^3^.

Consequently, episodic recall is an important element of the preclinical Alzheimer’s cognitive composite (PACC5)^4,5^, the aim of which is to provide a comprehensive assessment of AD-relevant cognitive impairment and to serve as a tool with validated sensitivity to detect cognitive decline over time^4,5^. However, traditional paper-and-pencil assessments to derive composites such as the PACC5 are time-consuming and require supervision by a trained neuropsychologist^5^. This severely restricts their utility and implementation in primary care, especially when considering equal opportunities for comprehensive neuropsychological assessments also in rural areas and the use of high-frequency cognitive assessment to account for day-to-day variations in cognitive performance. In general, the long test duration and specialized supervision make the high-frequency longitudinal use of established neuropsychological assessments practically impossible. There is, thus, a strong need for unsupervised, remote, high-frequency cognitive assessment that can provide meaningful approximation of traditional neuropsychological scores.

Mobile devices such as smartphones and tablets have the potential to enable unsupervised and remote high-frequency cognitive assessments^6–8^. Indeed, recent studies have demonstrated the feasibility of remote digital cognitive assessments in the general population^9^ as well as in older adults and patient populations^10–15^. Implementing a mobile and remote proxy for a neuropsychological assessment (such as the tests that comprise the PACC5) also offers the opportunity to overcome some of the shortcomings of neuropsychological tests. One potential disadvantage of established neuropsychological assessments of episodic memory is, for example, that they heavily tax verbal abilities, which makes it difficult to assess episodic memory in multi-lingual settings or when verbal abilities are already impaired^3^. In addition, implementing new cognitive tests allows us to take into account the latest insights into the functional architecture of episodic memory and the spread of AD pathology. Recent work on the functional neuroanatomy of episodic memory showed that episodic memory involves a network including medial temporal, midline parietal, and cortical regions, each of which serves different functions and is affected in different stages of AD^16,17^. Episodic memory requires pattern separation processes that are mediated by the dentate gyrus^18,19^ and reduce memory interference between similar events, and pattern completion processes that are mediated by hippocampal Cornu Ammonis 3 (CA3) and enable the recollection of details from a past event in interplay with neocortical regions^20^. A third aspect of episodic memory is recognition memory^21,22^. The medial temporal lobe regions provide information to the hippocampus mainly through the entorhinal cortex, where object and scene information is processed in partly segregated pathways^23–27^, and there is converging evidence that in addition to the long-term recall, short-term mnemonic discrimination of object and scene representations is impaired in the predementia stages of AD^28^.

**Fig. 1:**
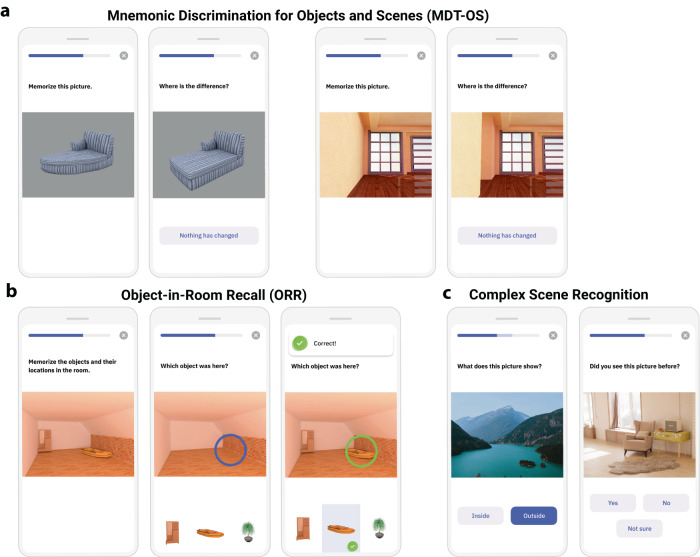
Memory tests constituting the RDMC score. **a** Mnemonic discrimination test for objects and scenes (MDT-OS). **b** Object-in-room-recall (ORR) test. **c** Complex scene recognition test (CSR).

A set of anatomically informed and non-verbal tasks for episodic memory that incorporate these recent insights into the functional anatomy of episodic memory is available on the neotiv digital platform (https://www.neotiv.com/en)^9,14^ and has been implemented in prospective cohort studies of the German Center for Neurodegenerative Diseases (DZNE) and the Wisconsin Registry for Alzheimer’s Prevention (WRAP). There are three different tests of memory (see Fig. 1). First, a short-term mnemonic discrimination test tapping into pattern separation (MDT-OS), separately implemented for object and scene stimuli; second, a short- and long-term cued-recall test of object-scene associations tapping into pattern completion (ORR) and, third, a long-term photographic scene recognition memory test (CSR).

Here we evaluate these three memory measures in a remote and unsupervised study design using mobile devices. To that end, we develop a remote digital memory composite (RDMC) score and assess its construct validity using in-clinic neuropsychological assessment as well as its retest reliability across independent test sessions. Finally, we assess the diagnostic accuracy of the RDMC score when differentiating between individuals with and without cognitive impairment in a memory clinic sample.

## Results

### Participant characteristics and recruitment

**Fig. 2:**
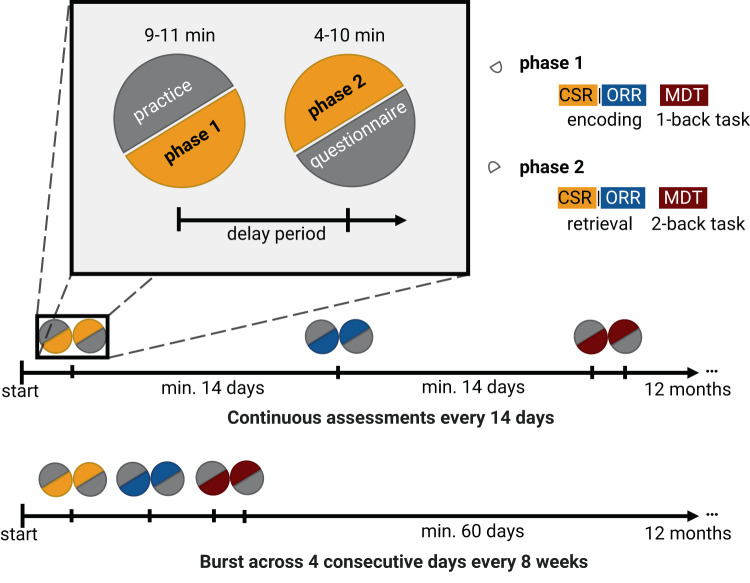
Timeline of the study protocol. Participants enlisted in a 12-month remote memory assessment study. During the initial session, subjects gave consent to participate in the study and installed the mobile app. For each session, they completed a short tutorial, followed by phase 1 of their respective task: encoding for ORR and CSR, and 1 back task for MDT-OS. Following the delay period, participants were notified that the next phase was available and could perform it immediately or postpone it if testing was inconvenient. Phase 2 consisted of retrieval for ORR and CSR and 2-back task for MDT-OS. It was followed by ratings regarding concentration, subjective performance, and distraction during the task. While most participants were notified regarding the next available test session every other week (continuous design), a subgroup within the WRAP cohort performed all three paradigms in a burst on four consecutive days every eight weeks (burst design).

Here, we considered 199 study participants who completed at least one session of each of the three cognitive tests (97 HC, 59 SCD, and 43 MCI; see Fig. 2 and “Materials and methods” for details regarding the study protocol). Following quality assurance as described in the “Materials and methods”, 143 individuals could be included who contributed at least one valid test session per paradigm.

We calculated a preclinical Alzheimer’s cognitive composite (PACC5) cut-off score across all participants without dementia in the entire DELCODE and WRAP cohorts baseline assessment, respectively, that distinguished MCI from cognitively unimpaired participants (HC and those individuals with subjective cognitive decline (SCD)) prioritizing sensitivity >0.8, and resulting in a cut-off of −0.515 for DELCODE and −0.981 for WRAP. In order to increase the comparability between cohorts and ensure the use of the most recent information on participant’s cognitive status, cognitive impairment was then defined as either an MCI diagnosis (at the baseline or follow-up assessment) or a PACC5 score suggestive of mild cognitive impairment at the closest follow-up session to the app-based assessment (below the derived cut-off for DELCODE and WRAP). This resulted in 105 cognitively unimpaired (CU) and 38 cognitively impaired participants (CI; see Table 1 for participants’ characteristics and Supplementary Table 2 for a comparison of cohorts). There was no statistical difference in RDMC performance between cognitively unimpaired individuals without subjective cognitive decline across the cohorts (*M*_DC/MC_ = −0.11, *M*_WRAP_ = 0.15, *t*(49.3) = −1.57, *p* = 0.123), nor was there a significant difference in PACC5 performance (*M*_DC/MC_ = 0.42, *M*_WRAP_ = 0.3, *t*(70) = 0.86, *p* = 0.395).

**Table 1:** Participant demographics

	HC (*N* = 78)	SCD (*N* = 40)	MCI (*N* = 25)	CU (*N* = 105)	CI (*N* = 38)	Total (*N* = 143)
Age (years)	68.2 (5.47)	69.2 (7.67)	69.2 (6.79)	67.9 (6.01)	70.7 (6.92)	68.6 (6.36)
Education (years)	15.9 (2.82)	14.6 (2.64)	13.9 (2.49)	15.7 (2.79)	13.9 (2.55)	15.2 (2.82)
Sex (female)	67.9%	52.5%	36%	62.9%	44.7%	58%
MMSE	29.5 (0.72)	29.4 (0.9)	26.9 (2.74)	29.6 (0.69)	27.5 (2.41)	29 (1.64)
RDMC	0.01 (0.67)	−0.21 (0.6)	−1.03 (0.69)	0 (0.65)	−0.87 (0.66)	−0.23 (0.76)

Abbreviations: *N* number of participants, *HC* Healthy Controls, *SCD* subjective cognitive decline, *MCI* mild cognitive impairment, *MMSE* mini-mental state examination, *RDMC* remote digital memory composite, *CU and CI* cognitively (un)impaired based on clinical diagnosis or the most recent PACC5. Displays mean values (standard deviations) unless otherwise stated.

### Contextual factors

Across all three cognitive tests, participants reported high concentration levels during the task (mean = 3.99, scale 1–5, which translates to good concentration) and moderately high subjectively rated task performance (mean = 3.51, scale 1–5, which translates to middling subjectively rated performance). While concentration levels were similar across tasks (3.64, 4.08, 4.23 for MDT-OS, ORR, and CSR, respectively), subjective performance indicated higher task difficulty for the MDT-OS (2.9) compared to ORR and CSR (3.66 and 3.97, respectively). In addition, participants reported no distractions during their test sessions in 92% of the tasks (MDT-OS: 90%, ORR: 92%, CSR: 96%).

Following filtering, the time between encoding and retrieval in the ORR and CSR tests was, on average, 67 min (SD = 36) and 92 min (SD = 23), respectively. In DELCODE, participants were invited to the retrieval phase of the ORR after 30 min, and their actual mean delay was 49 min, while they were invited to the CSR retrieval after 65 min and completed it on average after a delay of 82 min. In WRAP, participants were invited to the retrieval phases of both paradigms after 90 min and completed it on average after 97 min and 101 min for the ORR and CSR tasks respectively. Mobile devices had a screen diagonal between 10.2 and 32.9 cm (mean 16 cm, SD = 5.5), indicating the use of smartphones as well as tablet computers.

### Development of the remote digital memory composite

First, we built the RDMC using equal weights where each component (each of the three cognitive tests) had the same weight. While the CSR provides a corrected hit rate from the delayed recall, the ORR and the MDT provide two outcomes. In the ORR, there is an immediate as well as a delayed recall, while the MDT comes in two task conditions, one corrected hit rate for scenes and one for objects^9,14^. All individual components (ORR-Im, ORR-Del, MDT-O, MDT-S, and CSR) were z-standardized using the mean and standard deviation of the CU participants. For the RDMC, we then calculated the z-scored mean across ORR-Im and ORR-Del (TotalRecall), the z-scored mean score across MDT-O, MDT-S (TotalCorrectedHitRate), and the z-scored corrected hit rate of the CSR. The resulting three z-scores were averaged to derive the final RDMC score. The retest reliability between two independent time points was good (*r* = 0.8, *p* < 0.001, 95% CI [0.73–0.86]; ICC = 0.8), while the retest reliability of individual elements of the composite was in the moderate range (MDT-OS: *r* = 0.61, *p* < 0.001, 95% CI [0.49, 0.72]; ICC = 0.6; ORR: *r* = 0.62, *p* < 0.001, 95% CI [0.49, 0.72]; ICC = 0.65; CSR: *r* = 0.66, *p* < 0.001, 95% CI [0.54, 0.75]; ICC = 0.64).

### Relationship between the remote digital memory composite and the PACC5

**Fig. 3:**
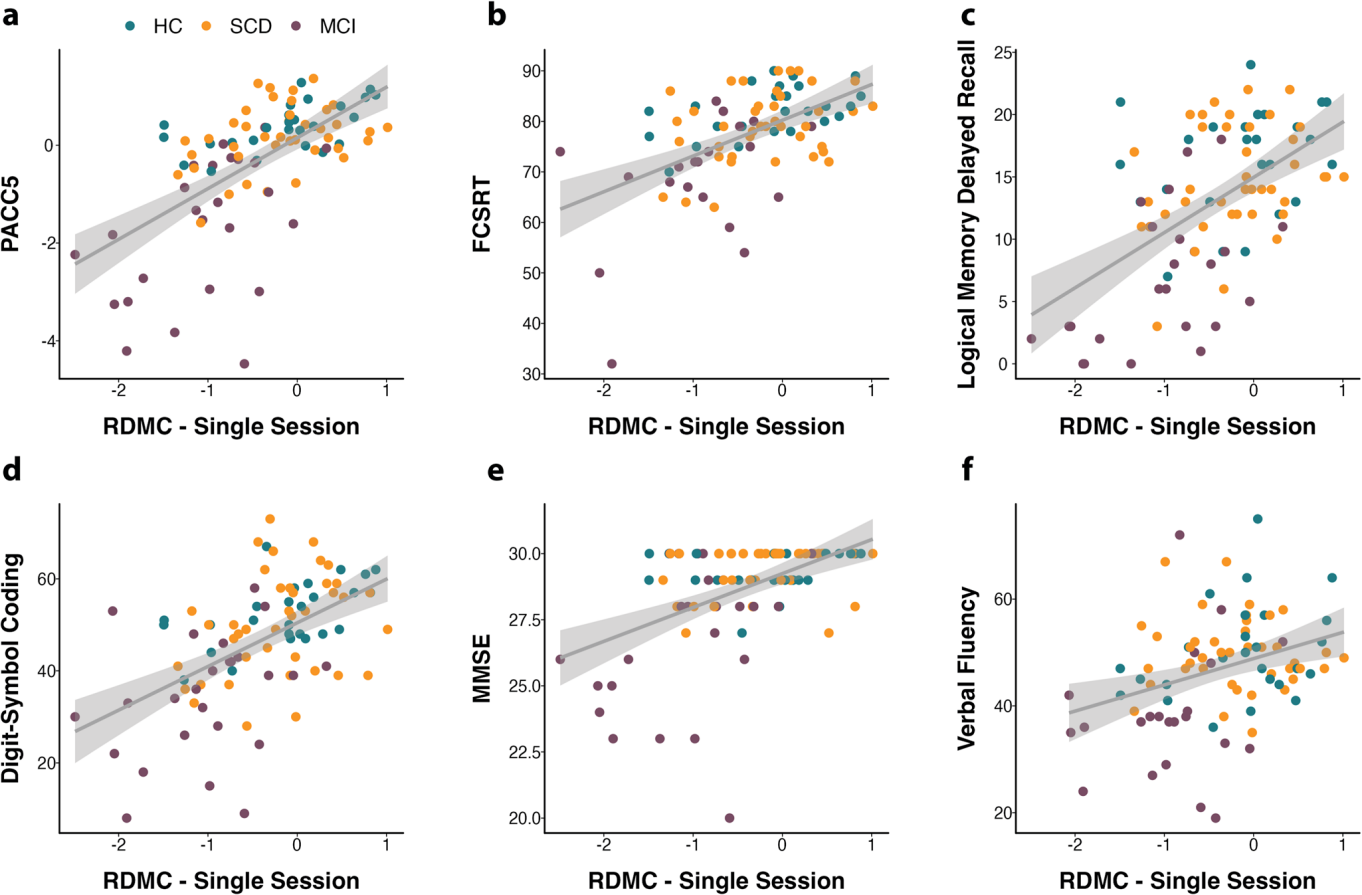
Construct validity of the composite score. Correlation between the RDMC (one session of each paradigm) and the closest-in-time **a** DELCODE PACC5 assessment as well as **b**–**f** all individual PACC5 elements. HC Healthy Controls, SCD subjective cognitive decline, MCI mild cognitive impairment, FCSRT free and cued selective reminding test, MMSE mini-mental state exam.

Next, we assessed the construct validity of the RDMC. To that end, we used neuropsychological data from the closest-in-time in-clinic visit in the DELCODE study (to the mobile app add-on study) and Memory Clinic to perform a correlation analysis between the RDMC and the PACC5 score as well as its individual elements. The average time interval between the in-clinic visits and the remote app assessments was 80 days. The RDMC correlated significantly (*r* = 0.62, *p* < 0.001) with the closest-in-time in-clinic PACC5 scores (see Fig. 3). When considering only participants with memory complaints, meaning those that were referred to the memory clinics by their general practitioner and fulfilled either SCD or MCI criteria, the construct validity of the RDMC remained good (*r* = 0.62, *p* < 0.001). The construct validity in individuals without memory complaints (HC) was similar (*r* = 0.59, *p* = 0.001). We used hierarchical multiple regression models to test whether each element of the RDMC (ORR-Total, MDT-OS, and CSR) added significantly to predicting the PACC5 score. Model comparisons using the Akaike information criterion (AIC) indicated a significant effect for each predicting component (see Supplementary Table 1). In addition, we assessed the relationship of the RDMC with each of the individual elements of the PACC5. The RDMC correlated strongest with the memory measures, the logical memory delayed recall (*r* = 0.55, *p* < 0.001), and the FCSRT96 (*r* = 0.53, *p* < 0.001) but also with the DSCT (*r* = 0.54, *p* < 0.001). As expected, we found weaker relationships with the MMSE (*r* = 0.49, *p* < 0.001) and verbal fluency (*r* = 0.34, *p* = 0.001). These relationships survived correction for multiple comparisons. In the WRAP sample, the RDMC showed a significant correlation with the PACC5 of *r* = 0.43 (*p* = 0.002). Finally, regarding known-groups validity, we observed a statistically significant difference in RDMC performance between healthy controls and individuals with subjective cognitive decline on the one hand (*M*_HC/SCD_ = −0.06) and MCI patients on the other hand (*M*_MCI_ = −1.03, *t*(33.7) = 6.5, *p* < 0.001) mirroring the group differences in the PACC5 (*M*_HC/SCD_ = 0.19, *M*_MCI_ = −1.69, *t*(26.8) = 6.4, *p* < 0.001.

### Relationship with age, sex, education and other factors

Next, we calculated multiple regression models with age, sex, years of education, time-to-retrieval, and screen size to identify the relationships with individual components of the RDMC. For CSR, only years of education was a significant predictor where higher education was associated with better performance (*β*_education_ = 0.12, *p* < 0.001; *β*_age_ = −0.007, *p* = 0.6; β_sex_ = −0.29, *p* = 0.1; *β*_delay_ = 0.19, *p* = 0.38; *β*_screen_ = −0.003, *p* = 0.839). For MDT-OS, only age was a significant predictor where higher age was associated with worse task performance (*β*_age_ = −0.027, *p* = 0.02; *β*_sex_ = −0.19, *p* = 0.204; *β*_education_ = 0.02, *p* = 0.464; *β*_screen_ = −0.02, *p* = 0.159). For the ORR, age and years of education were associated with task performance (*β*_age_ = −0.05, *p* < 0.001; *β*_education_ = 0.06, *p* = 0.049; *β*_sex_ = −0.1, *p* = 0.531; *β*_delay_ = 0.21, *p* = 0.139; *β*_screen_ = 0.009, *p* = 0.518). With respect to the RDMC, more years of education (*β*_education_ = 0.09, *p* = 0.002), as well as younger age (*β*_age_ = −0.04, *p* = 0.002), were associated with higher task performance, but not sex. In comparison, the PACC5 was associated with sex (*β*_sex_ = −0.66, *p* < 0.001), years of education (*β*_education_ = 0.09, *p* < 0.001), and age (*β*_age_ = −0.03, *p* = 0.03), i.e., women and participants with more years of education and those that were younger obtained a higher PACC5 score.

### Diagnostic accuracy of the remote digital memory composite

**Fig. 4:**
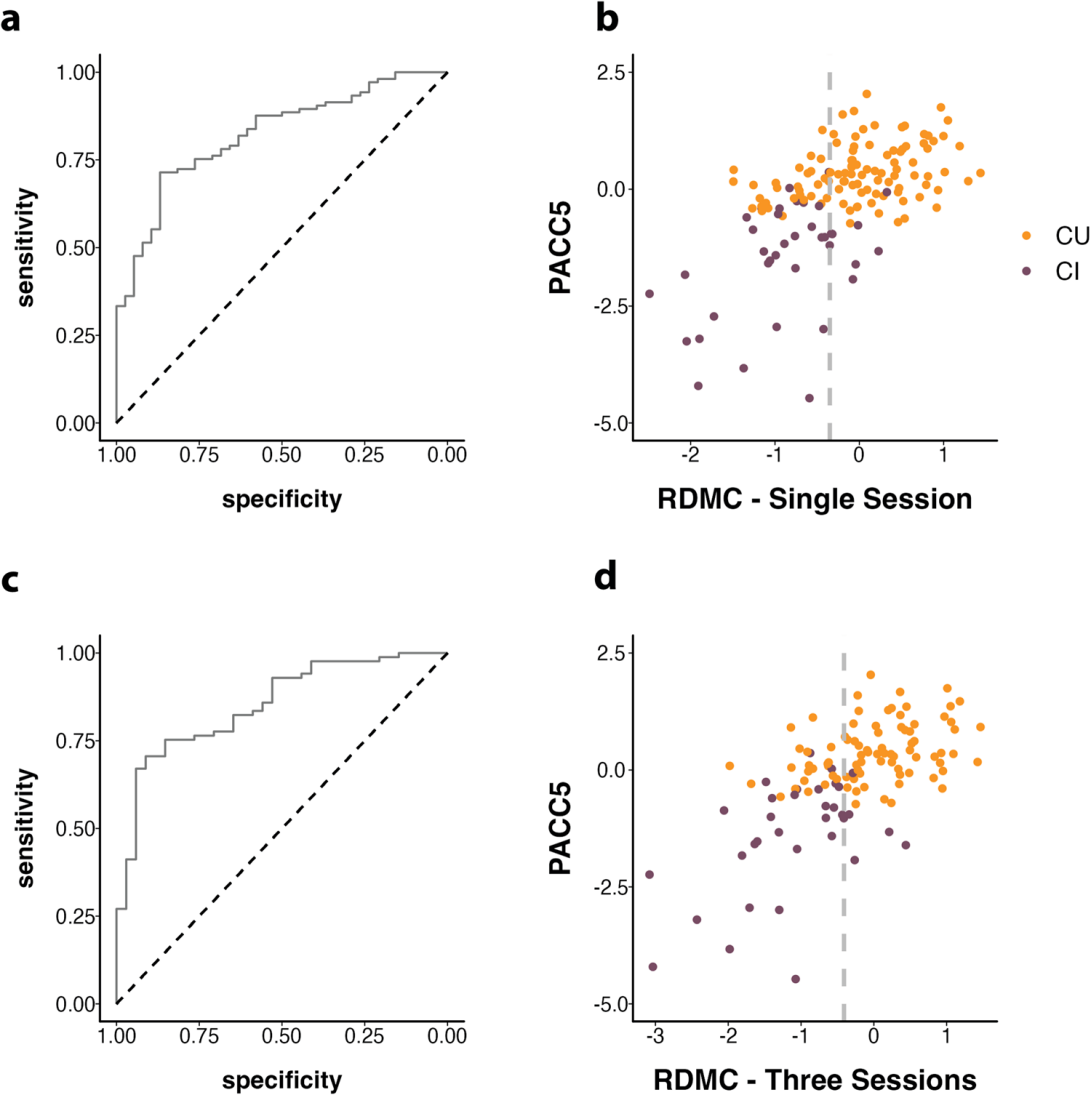
Diagnostic accuracy across Single Session and Three Session scenarios. **a**, **c** Receiver operating characteristic (ROC) curve showing diagnostic accuracy for the detection of MCI-grade cognitive impairment based on the RDMC. **b**, **d** Scatter plot showing the optimal RDMC cut-off with CU and CI in different colors—optimal cut-offs at −0.35 and −0.41 indicated by the dashed gray line, dots to the left are classified as cognitively impaired, and dots to the right as cognitively unimpaired. Information is shown for the single-session scenario (i.e., one session of each paradigm) and the three-session scenario.

Finally, we assessed the diagnostic accuracy of the RDMC. The RDMC score differentiated CI and CU individuals with a cross-validated Area under the Curve (cvAUC) of 0.83 [CI 0.66; 0.99], resulting in a sensitivity and specificity of 0.82 and 0.72 respectively (optimal cut-off = −0.35). The ROC curve and classifications with the cut-off are presented in Fig. 4A. Alternatively, considering only MCI patients or only participants with a PACC5 score below the MCI-grade cognitive impairment cut-off as cognitively impaired yielded very similar cvAUCs of 0.85 [CI 0.66; 0.99] and 0.84 [CI 0.67; 0.98] respectively. In order to test whether all three components of the RDMC are needed to achieve the best possible classification, we performed individual AUC analyses for each individual component (ORR = 0.78; MDT-OS = 0.78; CSR = 0.72) as well as for alternative composite scores covering all possible combinations of only two test paradigms (ORR/MDT-OS: = 0.82; ORR/CSR = 0.8; MDT-OS/CSR = 0.79). No individual component or composite combining two components could however reach an AUC of 0.83.

In addition, we used logistic regression models to test how a model containing the RDMC would compare to a model containing only participant age, sex, and years of education. The model that included the RDMC as a predictor (Tjur’s *R*^2^ = 0.325; AIC = 126.4) fit the data significantly better than the basic model that included participants age, sex, and years of education (Tjur’s *R*^2^ = 0.160; AIC = 151.4) and outperformed its diagnostic accuracy (AUC: 0.85 compared to AUC: 0.74). A maximum sensitivity of 84% could be achieved given at least 70% specificity, and a maximum specificity of 85% could be achieved given at least 70% sensitivity. For an additional scenario with up to three individual test sessions per memory test, 119 participants (85 CU and 34 CI) could be included (see Supplementary Table 3 for baseline characteristics). In this scenario, the RDMC score differentiated CI and CU individuals with a cross-validated cvAUC of 0.87 [CI 0.7; 0.99] and a sensitivity and specificity of 0.85 and 0.75 respectively (optimal cut-off = −0.41). The ROC curve and classifications with the cut-off are presented in Fig. 4C.

## Discussion

We developed an unsupervised, self-administered, and remote digital memory composite based on one test session from each of three equally weighted memory tests (ORR, MDT-OS, and CSR), which were performed remotely and fully unsupervised. The resulting RDMC showed good construct validity in relation to the PACC5 score, especially with its elements measuring memory, and good retest reliability in a subsample that performed each test twice. Finally, the RDMC could differentiate between individuals with and without MCI-grade impairment with an AUC of 0.83, demonstrating high diagnostic accuracy. An additional scenario with up to three individual test sessions per memory test with the aim of providing redundancy to account for potential distractions or interruptions while self-administrating the tests yielded an AUC of 0.87.

In terms of construct validity, we found a strong correlation between the RDMC and the PACC5, as well as its individual elements. This correlation was present in both cognitively healthy older adults and those with cognitive impairment, indicating that the correlation was not driven by collating an impaired and a non-impaired group as two extremes into the same analysis. The fact that the correlation also held within those with cognitive symptoms (SCD and MCI) and that all of these individuals were recruited on the basis of referrals (as opposed to recruitment advertisements) provides the first evidence that may support construct validity in a healthcare setting. In addition, we observed known groups validity. In terms of reliability, we found a high correlation between two different instances of the RDMC.

The RDMC identified individuals with an MCI-grade impairment with an AUC between 0.83 (single self-administration of each test) and 0.87 (three self-administrations of each test). This allowed us to identify individuals with MCI-grade impairment with a sensitivity of 0.82 and a specificity of 0.72 on the basis of a single assessment of the RDMC (three test sessions in total, one of each memory test). This is comparable to or higher than several other recently reported unsupervised^29^ or in-clinic and supervised digital cognitive assessments^30–33^. Importantly, however, several earlier approaches reported outcomes by comparing MCI patients against samples that exclusively consisted of healthy asymptomatic older adults^29–34^. In healthcare settings, the main challenge is to identify significant impairment within individuals with memory concerns. Therefore, we believe that our focus on individuals with memory complaints and the inclusion of a large number of individuals with subjective cognitive decline who sought medical advice (hence were not recruited through advertisements) in this sample is a major advantage in the validation and critical for future application.

Usability is a major limitation for mobile device-based assessment of cognition in old age, particularly in preclinical and prodromal AD. While participants were assisted during the installation of the neotiv mobile app and received a manual at the time when their consent was obtained at the memory clinic, all tests were conducted fully remotely and without supervision. Participants received a push notification on their mobile devices each time a test was available to be performed. All instructions and guidance for performing the tests were provided in the app and included a training run of each test. Participants were also instructed to seek a quiet place where they would not be distracted, and after each test, they were asked through a questionnaire whether they could perform the test without distraction. Our results show that participants were able to perform the tests in a distraction-free context, given that they reported no distraction in 92% of test sessions and, on average, high concentration levels. In addition, they reported, on average, medium-to-high subjective task difficulty across memory tests, indicating that while the remote cognitive assessments were challenging, they were neither too easy nor too difficult. Finally, the adherence to the mobile tests was quite good, with a maximum of 20% of participants dropping out after 6 tests within a period of at least 12 weeks. Our results, thus, indicate that it is possible to achieve the level of usability that is required to perform a detailed assessment of episodic memory fully remotely and without any supervision in a cohort of individuals with memory complaints. However, for future application in health care, it will be important to further address the digital divide among older adults as well as participants from underrepresented groups and those living in rural areas to take full advantage of the unprecedented opportunity to overcome the health care divide for Alzheimer’s disease offered by digital technologies. Recent data show that technology familiarity was not associated with study adherence in older adults and that older adults show remarkable adherence when thoughtful study planning emphasizes participant support and user-centered design^11^.

In order to obtain the RDMC, individual sessions of the ORR, CSR, and MDT-OS are required where each test consists of two phases (i.e., six separate 5- to 10-min sessions on three different days). In principle, all three tests could be obtained within a single day. However, we decided not to enforce the shortest possible acquisition time. Instead, we decided to leverage the opportunities of mobile and unsupervised testing to achieve a more meaningful implementation. To that end, we stretched out the assessment over several weeks to enable a more representative sampling of memory performance over time and thereby be less vulnerable to day-to-day performance fluctuations. We used spaced testing to ease stress for the participants and eliminate potential implementation problems that would lead to worries and complaints by those participants who felt being tested on a bad day. In an additional scenario, we extended this approach and included even more individual test sessions per paradigm with the aim of increasing the robustness of the approach, which yielded a higher diagnostic accuracy. Thus, in either scenario, the RDMC reflects memory performance over a period of several weeks rather than a single day, something that would be very difficult to implement with a supervised testing approach.

Episodic memory tests such as the FCSRT^35^ and the other elements of the PACC5 place heavy demands on verbal abilities. This significantly challenges applicability in international trials or in conditions with mild language disorders (e.g., due to a vascular event or primary progressive aphasia)^3^. The three tests of the RDMC established here, however, are not dependent on verbal abilities such as naming, word-finding, or pronunciation and thereby facilitate testing across different dementia syndromes and subtypes of AD as well as in international comparisons. Furthermore, the RDMC shows no overlap with the PACC5 in terms of the paradigm and modalities tested so that there would be no interference with a memory-clinic or trial-based PACC5 or related neuropsychological assessment following case-finding. Finally, we did not observe significant sex differences in the RDMC, which contrasts with other neuropsychological assessments, including the PACC5 in our analyses.

In the implementation of the ORR and CSR tests that were used in this work, we did not strictly reinforce adherence to the planned retrieval delay intervals of these tests, which led some individuals to perform recall and recognition assessments after prolonged delays (e.g., more than 180 min). Given that the length of the delay period is associated with task performance, we excluded sessions with significantly extended delay periods. However, this led to a significant exclusion of participants in our study. Thus, future implementations of the RDMC and remote and unsupervised assessments of long-term memory, in general, should make it easier for participants and patients to integrate remote and repeated tests into their everyday lives. In particular, for delayed retrieval tasks, it must be possible for participants to complete the tests according to their own schedule so that they can easily accommodate the delay period, meaning they should have a clear and transparent understanding of when encoding and retrieval phases will be happening, how long those will take, and until when the tasks need to be completed. For the RDMC, these insights have already resulted in a redesigned app interface that highlights this information for the participant.

This study has a number of shortcomings. First, while our results are based on three cohorts from two different countries, this represents a single study with a modest sample size and thus needs to be cross-validated across independent cohorts. Second, while we could show evidence for limited relationships between the RDMC and sample demographics, a large and diverse normative sample is needed in order to calculate demographically adjusted scores for various covariates. Third, our sample size was not yet sufficient to assess the relationship between AD biomarkers and the diagnostic accuracy of biomarker-stratified subgroups. Finally, assessing three different memory domains and even repetitions of each domain is time-intensive for participants and, therefore, can also be viewed as a limitation. On the other hand, a major advantage of digital technologies is that time constraints that are particularly relevant for on-site, in-person assessments can be overcome with digital and remote technologies. Here, a trade-off needs to be found between harnessing this advantage of digital technologies and minimizing the time burden on patients.

Taken together, good construct validity and retest reliability of the RDMC score pave the way for implementing mobile app-based remote assessment in clinical studies as well as in health care. The current data indicate that the RDMC can facilitate case-finding whenever the main question is about an individual’s MCI-grade cognitive impairment. Future studies need to show whether repeated assessments of the RDMC over time will be sensitive to cognitive change.

## Methods

### Participants and neuropsychological assessments

DELCODE is an observational longitudinal memory clinic-based multicenter study in Germany, with participants aged 60 years and older, who either have mild dementia of the Alzheimer’s type, amnestic mild cognitive impairment (MCI), subjective cognitive decline (SCD), or are asymptomatic healthy controls (HC). The DELCODE study has been registered in the German Clinical Trials Register (DRKS00007966), retrospectively (04/May/2015). The detailed study design of DELCODE is reported elsewhere^36^. The remote mobile monitoring add-on study started in April 2019 after a separate approval by the ethical committees of each participating site (Ethics committee of the University Hospital Magdeburg and the Ethics committees of the Medical Faculties at the University of Bonn, Ludwig-Maximilians-University Munich, University of Tübingen, Rostock University, University of Göttingen and University of Cologne). The annual neuropsychological testing in DELCODE included the PACC5^4^ and other assessments reported in full elsewhere^36^. The PACC5 z-score was calculated as the mean performance z-score across the MMSE^37^, a 30-item cognitive screening test, the WMS-R Logical Memory Delayed Recall^38^, a test of delayed (30 min) story recall, the Digit-Symbol Coding Test (DSCT; 0–93)^39^, a test of memory, executive function and processing speed, the Free and Cued Selective Reminding Test–Free Total Recall (FCSRT96; 0–96)^40^, a test of free and cued recall of newly learned associations, and the Category Fluency Test, a test of semantic memory and executive function. The z-scores for the PACC5 elements were derived using the mean and standard deviation of HC and SCD from the baseline visit of the entire DELCODE study sample. A PACC5 composite score was calculated when at least three of its five components were available while making sure that at least the MMSE, one memory measure, and either category fluency or DSCT were included. In the DELCODE cohort, the clinical labels (HC, SCD, MCI) were established at the baseline assessment of each participant, and follow-up diagnoses were established in April 2021^41^. At the Memory Clinic of the University of Magdeburg, which is a DELCODE recruitment site, additional participants were prospectively recruited according to the DELCODE neuropsychological protocol. The recruitment criterion for these additional participants was clinically suspected MCI on the basis of progressive memory complaints.

WRAP is a longitudinal observational cohort study enriched with persons with a parental history of probable AD dementia^42^. The neotiv add-on study began in April 2021. Neuropsychological testing in WRAP included the PACC5^4^ and was completed every two years. The PACC5 z-score was calculated as the mean performance z-score across the MMSE^37^, a 30-item cognitive screening test, the WMS-R logical memory delayed recall^38^, a test of delayed (30 min) story recall, the digit-symbol coding test (DSCT; 0–93)^39^, a test of memory, executive function and processing speed, the Rey Auditory Verbal Learning Test Total Learning Trials (RAVLT-Total; 0-75)^43^, a test of verbal learning, and the Category Fluency Test (animals in WRAP, animals and food in DELCODE) a test of semantic memory and executive function. The z-scores for the PACC5 elements were derived using the mean and standard deviation of cognitively unimpaired participants from the baseline visit of the entire WRAP cohort.

### Study design

All participants except patients with dementia were eligible if they owned a smartphone or tablet with internet access that was technically suitable for the mobile app to be installed on and that they could operate on their own. In addition, participants with sensory or motor problems or with significant neurological illness that may interfere with the ability to complete tasks were not eligible.

DELCODE participants were asked at their regularly scheduled annual follow-up visit and memory clinic patients during their in-clinic visit whether they would like to participate in the add-on study. If they agreed, study personnel did lend support in installing the app from the respective app store on the participants’ own mobile devices, but participants received no further verbal instructions apart from a printed manual. In WRAP, those who met eligibility criteria were sent a recruitment letter and the consent form and were contacted over the telephone ~2 weeks thereafter and invited to participate. If interested, a telephone screening was completed to confirm eligibility. Following screening, the informed consent process was completed, and verbal consent was obtained from the participant. Instructions for how to download the app were emailed and followed up over the telephone to provide additional assistance on downloading the app if needed. Task instructions within the app were provided in German and English, respectively.

### Remote and unsupervised cognitive tests

The object-in-room recall test (ORR), the mnemonic discrimination test for objects and scenes (MDT-OS), and the complex scene recognition test (CSR) were completed by participants remotely and unsupervised using their mobile devices. In all cohorts, participants were asked to complete memory assessments every two weeks (see Fig. 1). In WRAP; there was also an additional subgroup of participants who were asked to complete a burst of the same number of memory tasks every other month over four consecutive days. Each assessment consisted of a 2-phase session separated by a short delay. The two phases were either two halves of mnemonic discrimination or encoding and retrieval phases of complex scene recognition and object-in-room recall (see details of the tasks below). Every phase took around 5–10 min. Tests were remotely initiated via push notifications, which were sent at the same time of day as the registration, but participants had the possibility to postpone test sessions. This approach was chosen not to urge participants to take the test under suboptimal conditions such as distraction, fatigue or temporary illness. Daily reminders were sent via push notifications until the respective task was completed, and the actual time of testing was recorded. Before each test session, participants were reminded by the app to perform the test in a quiet environment, to put their glasses on if needed, and to ensure that their screen was bright enough to see the pictures clearly. They also received a short practice session at the beginning of each session. After each test session, participants were asked within the app if they were distracted by things happening around them during the session (yes/no decision) and to rate their concentration level and subjective performance (1 = very bad, 2 = bad, 3 = middling, 4 = good and 5 = very good). Hence, participants received the instructions for the cognitive tests remotely and performed the test fully unsupervised.

Figure 2A shows the outline of the MDT-OS test^23,24,26,44^. In this test, participants are presented with 3D-rendered computer-generated objects and scenes that are repeated either identically or in slightly modified versions. Participants need to decide whether a repeated presentation shows a repetition of the original picture or a modified version. They indicate their response by either tapping on a button (for an exact repetition) or tapping on the location of a change (for a modified version). They see 32 object and 32 scene pairs where half are repeated or modified respectively. One session was split into two phases and completed on two different days. The first phase was presented as a one-back task, while the second phase was presented as a two-back task. The test provides a hit rate, a false alarm rate, and a corrected hit rate for both the object and scene condition. The averaged corrected hit rate across the scene and the object condition is used for the RDMC.

Figure 2B shows the outline of the ORR test (for a discussion of the principles of pattern completion on which this test is based see^20^). In this test, participants were asked to memorize 3D-rendered computer-generated rooms in which two 3D-rendered objects are placed. Participants recall which object was placed at a specific location cued by a colored circle in the empty room in an immediate recall test. They indicate their recall decision by tapping on one of three objects displayed below the empty room: the correct object for that location, the object that was also present in the room but at a different location (correct source distractor), and a completely unrelated object (incorrect source distractor). They learn 25 such object-scene associations. After a delay of 30 min (DELCODE and Memory Clinic) or 90 min (WRAP), participants are prompted to complete a delayed recall test. However, as can be seen in the results section, the actual delay period based on the real-time test completion was similar across cohorts. In the ORR test, the ability to recall the correct association is graded and allows the separation of correct episodic recall from incorrect source memory. Thus, correct recall excludes the choice of an object that was present in the same room but at a different location (wrong source memory for specific location) and an object that was not present in the room but nevertheless associated with another room during encoding (wrong source memory for overall location). The test provides an immediate (0–25) and a delayed recall score (0–25). For the RDMC, we use the total recall, which is calculated as the average of the z-standardized immediate and delayed recall score.

Figure 2C shows the outline of the CSR test^21,22,45^. Participants see 60 photographic images depicting indoor and outdoor scenes. For encoding, participants make a button-press decision whether the presented scene is indoors or outdoors. After an instructed delay of 65 (DELCODE) or 90 (WRAP) minutes, the participants are informed via push notification to complete the second phase of the task. Here, the encoded images are presented together with 30 new images, and participants make old/new/uncertain recognition memory decisions. The test provides a hit rate, a false alarm rate, and a corrected hit rate. The corrected hit rate is used for the RDMC.

### Data handling

Participants used the app with a pseudonymized ID (no identifying information or clinical information was available or required in the mobile app) provided to them during an in-clinic visit or over the telephone. The app data were transferred to the research centers in accordance with the General Data Protection Regulation. The mobile app data were then related to the clinical data by the research centers and, in the following, released to Principal Investigators and to neotiv GmbH. Data handling and quality control procedures for the clinical DELCODE data are reported elsewhere^36^.

### Quality control procedures

For this manuscript, we analyzed data up until the data release on July 7th, 2022, in DELCODE and March 3rd, 2022, in WRAP. Recruitment of participants in the Memory Clinic Magdeburg was performed between October 2020 and January 2022. Regarding the sessions from the first wave, meaning each very first assessment of CSR, MDT-OS, and ORR, 8% of test sessions exceeded the threshold for missing responses (maximum of 20%), and 26.5% of test sessions exceeded the maximum length of the delay period (180 min) before filtering. Thus, these sessions were excluded during the quality assessment. Excluded test sessions were replaced by valid subsequent sessions where possible. As a result, 82% of the test sessions we report here were from the first wave and 18% from subsequent test sessions.

### Statistical analysis

All statistical analyses were performed in R (version 4.1.2, R Core Team, 2020). We correlated the RDMC score with in-clinic neuropsychological assessments to assess construct validity using the Pearson correlation coefficient, and results were corrected for multiple comparisons using Bonferroni correction. We also assessed known groups’ validity using an independent-sample-*t*-test. Multiple regression models were used to assess the relationship between age, sex, and years of education on the PACC5 as well as the RDMC. We conducted these analyses primarily within the sample from the DELCODE study and Memory Clinic of the University of Magdeburg, given that a PACC5 score with slightly different elements was used in the WRAP cohort. For further validation, we also report the correlation of the RDMC and the PACC5 in the WRAP cohort. Hierarchical multiple regression models were used to test whether each element of the RDMC (ORR-Total, MDT-OS, and CSR) contributed significantly to predicting the PACC5 score. Model comparisons using the Akaike Information Criterion (AIC) were used to test for significance. A lower value of AIC indicates a better fitting model, and a reduction ≥2 was considered significant. In addition, we assessed the influence of the time-to-retrieval and the screen size of the mobile device on the individual components of the RDMC as well as on the RDMC itself. We also assessed retest reliability across a time interval of 9 weeks (SD = 5.9 weeks) using the Pearson Correlation Coefficient and the Intraclass Correlation Coefficient. Diagnostic accuracy and receiver operating characteristic (ROC) analyses were performed using the pROC package^46^. A cross-validated Area under the Curve (cvAUC) was derived from 5-fold cross-validation. Logistic regression models, including participant age, sex, and years of education, were used to compare models containing the RDMC to models only containing demographic information. Regarding potential challenges in broader use within health care, we analyzed an additional scenario that would improve reliability and robustness above and beyond the provision of a single test. While the RDMC was calculated using one session of each memory test, for this scenario, we considered four repetitions of each test where the worst test performance of each paradigm was removed. This provides redundancy to account for distractions and interruptions that might appear and allows averaging across up to three test sessions to increase the representativeness of the overall test result. Therefore, we only included participants who provided up to four valid test sessions of each paradigm, removed each participant’s worst performance per paradigm, calculated a mean score for each outcome and the respective z-score regarding the mean and standard deviation of the cognitively unimpaired group before we calculated a composite by averaging across the three resulting means.

### Reporting summary

Further information on research design is available in the Nature Research Reporting Summary linked to this article.

### Supplementary information

Supplemental Material

Reporting Summary

## Data Availability

Data, study protocol, and biomaterials can be shared with partners based on individual data and biomaterial transfer agreements.
