# Supplementary material for: A remote digital memory composite to detect cognitive impairment in memory clinic samples in unsupervised settings using mobile devices
Source: NPJ Digit Med. 2024 Mar 26;7:79. doi: 10.1038/s41746-024-00999-9 (PMC10965892; doi:10.1038/s41746-024-00999-9)
Supplement: Supplementary file 1 — Supplemental Material [file 41746_2024_999_MOESM1_ESM.docx]

A Remote Digital Memory Composite to Detect Cognitive Impairment in Memory Clinic Samples in Unsupervised Settings using Mobile Devices

David Berron^1,2,3^, Wenzel Glanz^1,4^, Lindsay Clark^5,6^, Kristin Basche^5^, Xenia Grande^1,4^, Jeremie Güsten^1,4^, Ornella V. Billette^3^, Ina Hempen^3^, Muhammad Hashim Naveed^3^, Nadine Diersch^3^, Michaela Butryn^1,4^, Annika Spottke^7^, Katharina Buerger^8,9^, Robert Perneczky^8,10,11,12^, Anja Schneider^7,13^, Stefan Teipel^14,15^, Jens Wiltfang^16,17^, Sterling Johnson^5,6^, Michael Wagner^7,13^, Frank Jessen^18^, Emrah Düzel^1,4,3^

^1^ German Center for Neurodegenerative Diseases, Magdeburg, Germany

^2^ Clinical Memory Research Unit, Department of Clinical Sciences Malmö, Lund University, Lund, Sweden

^3^ neotiv GmbH, Magdeburg, Germany

^4^ Institute for Cognitive Neurology and Dementia Research, Otto-von-Guericke University, Magdeburg, Germany

^5^ Department of Medicine, Division of Geriatrics and Gerontology, University of Wisconsin, School of Medicine and Public Health, Madison, Wisconsin, US

^6^ Geriatric Research Education and Clinical Center, William S. Middleton Memorial Veterans Hospital, Madison, Wisconsin, US

^7^ German Center for Neurodegenerative Diseases, Bonn, Germany

^8^ German Center for Neurodegenerative Diseases, Munich, Germany

^9^ Institute for Stroke and Dementia Research (ISD), University Hospital, LMU Munich, Munich, Germany

^10^ Department of Psychiatry and Psychotherapy, University Hospital, LMU Munich, Munich, Germany

^11^ Munich Cluster for Systems Neurology (SyNergy), Munich, Germany

^12^ Ageing Epidemiology Research Unit (AGE), Imperial College London, London, UK

^13^ Department for Neurodegenerative Diseases and Geriatric Psychiatry, University Hospital Bonn, Bonn, Germany

^14^ Department of Psychosomatic Medicine, Rostock University Medical Center, Rostock, Germany

^15^ German Center for Neurodegenerative Diseases, Rostock, Germany

^16^ German Center for Neurodegenerative Diseases, Göttingen, Germany

^17^ Department of Psychiatry and Psychotherapy, University Medical Center Göttingen, University of Göttingen, Göttingen, Germany

^18^ German Center for Neurodegenerative Diseases, Cologne, Germany

**Supplementary tables:**

Supplementary table 1:

|  | ORR | | | ORR + CSR | | | ORR + CSR + MDT-OS | | |
| --- | --- | --- | --- | --- | --- | --- | --- | --- | --- |
| Predictors | Estimate | 95% CI | *p*-value | Estimate | 95% CI | *p*-value | Estimate | 95% CI | *p*-value |
| (intercept) | 0.142 | [0.000, 0.284] | **0.050** | 0.165 | [0.025, 0.305] | **0.022** | 0.183 | [0.044, 0.322] | **0.010** |
| ORR \| Total Recall | 0.551 | [0.415, 0.687] | **<0.001** | 0.488 | [0.347, 0.631] | **<0.001** | 0.420 | [0.270, 0.571] | **<0.001** |
| CSR \| Corrected Hit Rate |  |  |  | 0.175 | [0.040, 0.311] | **0.012** | 0.132 | [–0.006, 0.270] | 0.061 |
| MDT-OS \| Corrected Hit Rate |  |  |  |  |  |  | 0.215 | [0.037, 0.393] | **0.019** |
| Observations | 143 | | | 143 | | | 143 | | |
| R^2^ / R^2^ adjusted | 0.312 / 0.307 | | | 0.343 / 0.333 | | | 0.368 / 0.355 | | |
| AIC | 357.367 | | | 352.850 | | | 349.124 | | |

Supplementary table 2:

|  | CU | | CI | | Total | |
| --- | --- | --- | --- | --- | --- | --- |
|  | DC/MC (N=59) | WRAP (N=46) | DC/MC (N=32) | WRAP (N=6) | DC/MC (N=91) | WRAP (N=52) |
| Age (years) | 67.4 (6.16) | 68.5 (5.81) | 70.5 (7.40) | 71.5 (3.71) | 68.5 (6.75) | 68.9 (5.66) |
| Education (years) | 14.7 (2.58) | 16.9 (2.54) | 13.7 (2.47) | 15.2 (2.86) | 14.3 (2.56) | 16.7 (2.61) |
| Sex (N and % female) | 38 (64.4%) | 28 (60.9%) | 13 (40.6%) | 4 (66.7%) | 51 (56.0%) | 32 (61.5%) |
| MMSE | 29.5 (0.774) | 29.7 (0.566) | 27.3 (2.54) | 28.7 (1.03) | 28.7 (1.93) | 29.5 (0.699) |
| RDMC | –0.117 (0.614) | 0.150 (0.671) | –0.965 (0.653) | –0.341 (0.408) | –0.415 (0.745) | 0.0937 (0.662) |
| Note. Means and standard deviations are reported unless otherwise indicated. | | | | | | |

Supplementary table 3:

|  | CU (N=85) | CI (N=34) | Total (N=119) |
| --- | --- | --- | --- |
| Age (years) | 67.3 (5.63) | 70.1 (7.03) | 68.1 (6.17) |
| Education (years) | 15.5 (2.62) | 14.1 (2.62) | 15.1 (2.69) |
| Sex (female) | 52 (61.2%) | 16 (47.1%) | 68 (57.1%) |
| MMSE | 29.6 (0.599) | 27.5 (2.42) | 29.0 (1.67) |
| RDMC | 0 (0.716) | -1.10 (0.817) | -0.313 (0.894) |
| Note. Means and standard deviations are reported unless otherwise indicated. | | | |
