# Supplementary material for: A remote digital memory composite to detect cognitive impairment in memory clinic samples in unsupervised settings using mobile devices
Source: NPJ Digit Med. 2024 Mar 26;7:79. doi: 10.1038/s41746-024-00999-9 (PMC10965892; doi:10.1038/s41746-024-00999-9)
Supplement: Supplementary file 2 — Reporting Summary [file 41746_2024_999_MOESM2_ESM.pdf]

nature portfolio

Corresponding author(s): David Berron
Last updated by author(s): Oct 5, 2023

Reporting Summary

Nature Portfolio wishes to improve the reproducibility of the work that we publish. This form provides structure for consistency and transparency in reporting. For further information on Nature Portfolio policies, see our Editorial Policies and the Editorial Policy Checklist.

Statistics

For all statistical analyses, confirm that the following items are present in the figure legend, table legend, main text, or Methods section.

n/a	Confirmed	
☐	☒	The exact sample size (*n*) for each experimental group/condition, given as a discrete number and unit of measurement
☐	☒	A statement on whether measurements were taken from distinct samples or whether the same sample was measured repeatedly
☐	☐	The statistical test(s) used AND whether they are one- or two-sided<br>*Only common tests should be described solely by name; describe more complex techniques in the Methods section.*
☐	☒	A description of all covariates tested
☐	☐	A description of any assumptions or corrections, such as tests of normality and adjustment for multiple comparisons
☐	☒	A full description of the statistical parameters including central tendency (e.g. means) or other basic estimates (e.g. regression coefficient) AND variation (e.g. standard deviation) or associated estimates of uncertainty (e.g. confidence intervals)
☐	☒	For null hypothesis testing, the test statistic (e.g. *F*, *t*, *r*) with confidence intervals, effect sizes, degrees of freedom and *P* value noted<br>*Give P values as exact values whenever suitable.*
☒	☐	For Bayesian analysis, information on the choice of priors and Markov chain Monte Carlo settings
☐	☒	For hierarchical and complex designs, identification of the appropriate level for tests and full reporting of outcomes
☐	☒	Estimates of effect sizes (e.g. Cohen's *d*, Pearson's *r*), indicating how they were calculated

Our web collection on statistics for biologists contains articles on many of the points above.

Software and code

Policy information about availability of computer code

Data collection	The neotivApp for iOS and Android was used in order to collect memory measures remotely from participants.
Data analysis	All statistical analyses were performed in R (version 4.1.2)

For manuscripts utilizing custom algorithms or software that are central to the research but not yet described in published literature, software must be made available to editors and reviewers. We strongly encourage code deposition in a community repository (e.g. GitHub). See the Nature Portfolio guidelines for submitting code & software for further information.

Data

Policy information about availability of data

All manuscripts must include a data availability statement. This statement should provide the following information, where applicable:

- Accession codes, unique identifiers, or web links for publicly available datasets
- A description of any restrictions on data availability
- For clinical datasets or third party data, please ensure that the statement adheres to our policy

Data, study protocol and biomaterials can be shared with partners based on individual data and biomaterial transfer agreements.

Research involving human participants, their data, or biological material

Policy information about studies with human participants or human data. See also policy information about sex, gender (identity/presentation), and sexual orientation and race, ethnicity and racism.

Reporting on sex and gender	Information on sex was acquired using self report and used as a covariate in the analyses throughout the paper.
Reporting on race, ethnicity, or other socially relevant groupings	There is no systematic collection of information on race or ethnicity across cohorts and they are not considered throughout the analyses in this paper.
Population characteristics	We use age, sex, years of education as well as clinical diagnosis for mild cognitive impairment.
Recruitment	Participants were recruited from a memory clinic or out of two already existing longitudinal cohort studies. Everyone except patients with dementia were eligible if they owned a smartphone or tablet with internet access that was technically suitable for the mobile app to be installed on and that they could operate on their own. This might have introduced a potential bias regarding technical familiarity.
Ethics oversight	Ethical committees of each participating site approved the study.

Note that full information on the approval of the study protocol must also be provided in the manuscript.

Field-specific reporting

Please select the one below that is the best fit for your research. If you are not sure, read the appropriate sections before making your selection.

☐ Life sciences ☒ Behavioural & social sciences ☐ Ecological, evolutionary & environmental sciences

For a reference copy of the document with all sections, see nature.com/documents/nr-reporting-summary-flat.pdf

Behavioural & social sciences study design

All studies must disclose on these points even when the disclosure is negative.

Study description	This was a cross-sectional quantitative observational study.
Research sample	The sample consists of male and female cognitively unimpaired research participants as well as individuals with subjective cognitive decline and mild cognitive impairment who presented in a memory clinic.
Sampling strategy	Participants were recruited from a memory clinic or out of two already existing longitudinal cohort studies. Everyone except patients with dementia were eligible if they owned a smartphone or tablet with internet access that was technically suitable for the mobile app to be installed on and that they could operate on their own. This might have introduced a potential bias regarding technical familiarity.
Data collection	Cognitive data was collected via participants' mobile devices using the neotivApp. Clinical data was collected using paper-pencil assessments, clinical anamnesis and self reports.
Timing	For this manuscript, we analyzed data from April 2019 until the data release on July 7th 2022 in DELCODE and from April 2021 to March 3rd 2022 in WRAP. Recruitment of participants in the Memory Clinic Magdeburg was performed between October 2020 and January 2022.
Data exclusions	Regarding the sessions from the first wave, meaning each very first assessment of CSR, MDT-OS and ORR, 8% of test sessions exceeded the threshold for missing responses (maximum of 20%) and 26.5% of test sessions exceeded the maximum length of the delay period (180 minutes) before filtering.
Non-participation	We did not track systematically the reasons for people not participating in the study.
Randomization	Participants were not allocated in different experimental groups.

Reporting for specific materials, systems and methods

We require information from authors about some types of materials, experimental systems and methods used in many studies. Here, indicate whether each material, system or method listed is relevant to your study. If you are not sure if a list item applies to your research, read the appropriate section before selecting a response.

Materials & experimental systems

n/a	Involved in the study	
☒	☐	Antibodies
☒	☐	Eukaryotic cell lines
☒	☐	Palaeontology and archaeology
☒	☐	Animals and other organisms
☐	☒	Clinical data
☒	☐	Dual use research of concern
☒	☐	Plants

Methods

n/a	Involved in the study	
☒	☐	ChIP-seq
☒	☐	Flow cytometry
☒	☐	MRI-based neuroimaging

Clinical data

Policy information about clinical studies

All manuscripts should comply with the ICMJE guidelines for publication of clinical research and a completed CONSORT checklist must be included with all submissions.

Clinical trial registration

Provide the trial registration number from ClinicalTrials.gov or an equivalent agency.

Study protocol

Jessen, F., Spottke, A. & Alzheimer's ..., B. H. Design and first baseline data of the DZNE multicenter observational study on predementia Alzheimer's disease (DELCODE). (2018).

Johnson, S. C. et al. The Wisconsin Registry for Alzheimer's Prevention: A review of findings and current directions. Alzheimer's & Dementia: Diagnosis, Assessment & Disease Monitoring 10, (2018).

Data collection

see above

Outcomes

does not apply
